# Development of an Early Embryo Detection Methodology for Quail Eggs Using a Thermal Micro Camera and the YOLO Deep Learning Algorithm

**DOI:** 10.3390/s22155820

**Published:** 2022-08-04

**Authors:** Victor Massaki Nakaguchi, Tofael Ahamed

**Affiliations:** 1 Graduate School of Science and Technology, University of Tsukuba, Tennodai 1-1-1, Tsukuba 305-8577, Japan; 2 Faculty of Life and Environmental Sciences, University of Tsukuba, Tennodai 1-1-1, Tsukuba 305-8577, Japan

**Keywords:** quail eggs, thermal imaging, precision livestock farming, embryo detection, YOLO, deep learning

## Abstract

Poultry production utilizes many available technologies in terms of farm-industry automation and sanitary control. However, there is a lack of robust techniques and affordable equipment for avian embryo detection and sexual segregation at the early stages. In this work, we aimed to evaluate the potential use of thermal micro cameras for detecting embryos in quail eggs via thermal images during the first 168 h (7 days) of incubation. We propose a methodology to collect data during the incubation period. Additionally, to support the visual analysis, YOLO deep learning object detection algorithms were applied to detect unfertilized eggs; the results showed its potential to distinguish fertilized eggs from unfertilized eggs during the incubation period, after filtering radiometric images. We compared YOLOv4, YOLOv5 and SSD-MobileNet V2 trained models. The mAP@0.50 of the YOLOv4, YOLOv5 and SSD-MobileNet V2 was 98.62%, 99.5% and 91.8%, respectively. We also compared three testing datasets for different intervals of rotation of eggs, as our hypothesis was that fewer turning periods could improve the visualization of fertilized egg features, and applied three treatments: 1.5 h, 6 h, and 12 h. The results showed that turning eggs in different periods did not exhibit a linear relation, as the F1 Score for YOLOv4 of detection for the 12 h period was 0.569, that for the 6 h period was 0.404 and that for the 1.5 h period was 0.384. YOLOv5 F1 Scores for 12 h, 6 h and 1.5 h were 1, 0.545 and 0.386, respectively. SSD-MobileNet V2 performed F1 scores of 0.60 for 12 h, 0.22 for 6 h and 0 for 1.5 h turning periods.

## 1. Introduction

Precision approaches to improve yield are currently based on sophisticated support decision systems that include several types of sensors, powerful processing units for multidimensional data analysis and machinery automation. For animal production, certain authors considered that combining precision yield techniques with intensive farming systems constitutes the best way to enhance productivity and sustainability [1,2]. Therefore, the best practices to save natural resources include the mitigation of losses and the systematic use of energy inside the bioproduction ecosystem.

On breeding farms, the critical processes of genetic improvement, incubation and hatching are processed. This type of farm is responsible for providing specialized strains to other farms (meat and egg production) and even for supplying the pharmaceutical industry during vaccine research and manufacturing [3]. Historically, the insufficiency of efficient, affordable, and robust technologies has driven these farms toward low-precision hatching and culling of undesirable strains. In conventional hatching, there is a lack of rapid, high-confidence methods to identify unfertilized eggs and dead embryos in early stages. Due to this low-precision hatching, hidden costs are associated with a waste of energy and physical space during incubation periods [4]. For culling undesirable genes, there is also a deficiency of real-time methods and industrial equipment for sex segregation at the embryo level.

The most common technique for embryo detection is still candling, which consists of using a light source against the eggshell to view the content inside the eggs; nevertheless, it represents an inefficient, labor-consuming and subjective technique [5]. Moreover, candling is not applied to quail eggs because their small size and different eggshell colors render its utilization difficult. Several nondestructive methods to assess chicken egg content in early stages have been reported, including visible light transmission change detection [6], acoustic resonance [7], near-infrared (NIR) hyperspectral imaging [8], spectroscopy methods using visible (VIS)/NIR [9,10], optical sensing using PhotoPlethysmoGraphy (PPG) and deep learning classification [3].

Quail farms are well distributed worldwide as are chicken farms, although the largest producers of eggs are concentrated in East Asia and Brazil [11]. The consumption of this product represents approximately 10% of all eggs that are globally consumed [12]. Furthermore, the production of quail for meat and eggs has been increasing as the global demand for food continues to grow. In developing countries, quail poultry represents a viable alternative to supply animal protein, especially because of the reduced size of birds, the high nutritional value and the resilience of these avians for raising in “backyard” systems [13]. However, as quail farms have not yet been industrially established in many countries, breeding programs are not easily identified; therefore, farmers are responsible for breeding their own flocks, which is an arduous task that can lead to genetic depression of quail flocks caused by consanguinity, resulting in low fertility, low productivity of eggs and a high rate of mortality [14]. Another recurrent problem associated with quail egg production is the low hatchability rate; on average, 40% of all incubated eggs do not hatch. Several factors can contribute to this problem, the most common being long-term storage under bad climate conditions, dead embryos, or unfertilized eggs [13].

Recently, reliable methods using noncontact and nondestructive analysis in real time have come to represent the best opportunity for embryo detection in the poultry industry. The advent of big data, powerful processing units and more efficient algorithms are considered to be responsible for bringing computer vision (CV) methods using the deep neural network (DNN) approach to fruition. There are several types of heuristic algorithms based on neural networks (NNs), such as convolutional neural networks (CNNs). The ability of CNNs to address complex nonlinear problems, such as image recognition and classification, is responsible for presenting machines with vision sense and mimicking human’s capacity to solve problems. However, the greatest challenges for these algorithms concern speed and accuracy; sturdy models are those able to generalize predictions of any new data with a high confidence level similar to humans.

The You Only Look Once (YOLO) object detection algorithm is currently one of the fastest and most accurate models for image classification. The YOLO object detection algorithm was released by Redmon et al., 2016 [15]. The breakthrough of this algorithm was to use a single CNN to predict classes and bounding box coordinates as a regression problem. This algorithm can also be referred to as a Single Shot Detector in the class of one-stage detectors. Once an image is viewed by dividing it into a grid with a size of S × S, the algorithm predicts the class and bounding boxes for each grid cell. Two-stage object detector models, such as the R-CNN [16] series (including Fast R-CNN [17] and Faster R-CNN [18]), use the region proposal technique to divide the image into regions and then classify each region according to the proposal boxes. Although this technique effectively requires too much time for training, so it is not possible for real-time detection.

The main component of CV systems is the camera. Optical cameras capture light wavelengths in the visible light range of the electromagnetic spectrum; nevertheless, limitations arise due to light reflectance dependency. Thermal cameras can overcome light dependency once they capture radiometric information transmitted through the air by measuring the temperature of an object surface and by solving the intensity of infrared spectral wavelengths that reach the camera. The high-cost equipment, low resolution, reduced field of view and low frame recording represent the main limitations of thermal cameras [19]. However, the recently increasing demand associated with many applications, including healthy monitoring, is enabling thermal cameras to become popular sensors, which could make them more accessible in the near future.

In addition, the recent 5th generation (5G) network has enabled fast and long-range area coverage allowing rural areas connection to the internet. The main characteristics of this new generation of telecommunication network are ultralow latency, high reliability, energy efficiency and scalability [20,21], which were considered the main drawbacks for information and communication technology (ICT) implementation on farming 4.0 [22,23]. The 5G technology outperforms previous generations of 4G and 4G long-term evolution (LTE) by 100 times faster speed [24], Furthermore, it supports the internet of things (IoT) platforms with AI-based systems embedded into it, and, therefore, facilitates big data analysis and real-time intervention. As a consequence, 5G is contributing to the establishment of smart farming and remote management of poultry breeding farms.

The aim of this work was to investigate the potential of thermal micro cameras for fast visual, early embryo detection in quail eggs, as a supportive method to improve the hatching rate and to contribute to the further development of automatic incubator systems that are able to segregate fertilized and unfertilized eggs. Wild birds rotate their eggs several times a day during natural incubation to improve hatchability [25], and the rotation of eggs is essential to ensure normal embryo development in many avian species [26,27,28]. The hypothesis was that longer intervals between turning eggs could make the identification of unfertilized eggs easier using thermal-based visual detection systems. It was considered that less rotations could keep the developing embryo static and its temperature less distributed in the internal content, therefore facilitating interpretation by thermal cameras.

## 2. Materials and Methods

### 2.1. Thermal Imaging

**Figure 1:**
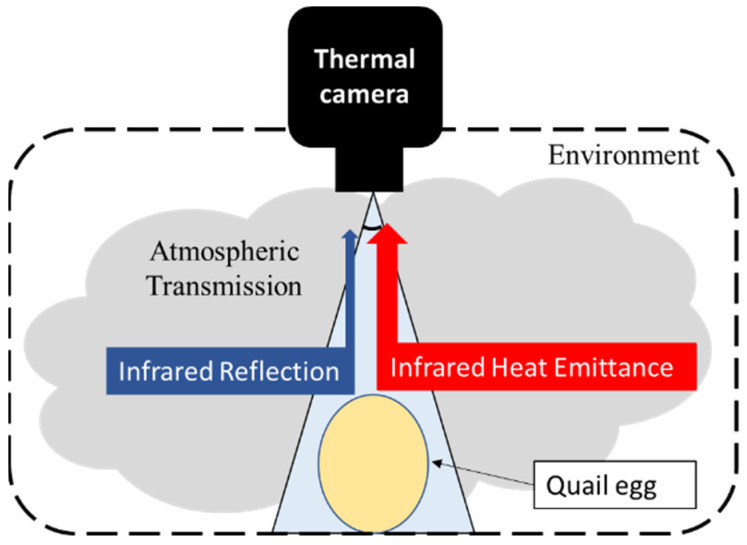
Thermal camera radiance relationship among material, environment, and the atmosphere.

As a noncontact nondestructive method, thermal imaging can be defined as the sum of the radiance emitted from a material, the environment (other material radiance) and atmospheric transmission (Equation (1)). Most of the challenges associated with thermal imaging center on interpreting absolute temperatures because thermal radiometric cameras only read information from opaque materials. Moreover, thermal imaging may be affected by the radiometric properties of objects and the medium: transmittance (τ), emissivity (ε) and reflectance (Figure 1), including the body itself and surroundings [29,30]. Another factor that influences the interpretation of radiometric images is the size of the target. Small surfaces may make the measurements difficult because the number of pixels describing the surface is diminished.

(1) $$\mathrm{Rcam}=\;\tau \varepsilon \mathrm{Rmat}+\;\tau \left(1-\varepsilon \right)\mathrm{Renv}+\left(1-\;\varepsilon \right)\mathrm{Ratm}$$

Rcam is the radiance read by the camera, Rmat denotes the radiance emitted by the material of interest or body, and Ratm is the radiance from the atmosphere.

#### 2.1.1. Transmittance (τ)

Transmittance is the ratio of a radiant flux transmitted (Փt) to an incident flux (Փi) [31] in function of the emission wavelength (λ). Atmospheric transmittance represents one of the greatest issues in thermal imaging analyses because it can change the radiometric temperature measurement, thus interfering with the active heat read by the camera (Equation (2)) Inside the incubator machines, humidity and temperature influence the transmission of radiance from the eggs, low humidity reduces the transmission of radiance, and the best strategy for enhancing the transmission in this case is to reduce the distance from the target.

(2) $$\tau \left(\lambda \right)=\frac{{Փ}_{t}\left(\lambda \right)}{{Փ}_{i}\left(\lambda \right)}$$

#### 2.1.2. Emissivity (ε)

Emissivity is the effectiveness of a material to emit thermal energy compared with a perfect absorbing energy body, a blackbody, at the same temperature. The real values are measured on a scale from 0 to 1. The emissivity depends on material characteristics, such as format, temperature, roughness, spectral wavelength, oxidation, and view angle [31]. The emissivity of the *Galloanserinae* species eggs (chicken, quail, turkey, duck, and swan) is higher, approximately 0.98 to 1 [32]. The emissivity (Equation (3)) can be defined as the absorption light of a blackbody minus the reflectance of the object ($R_{e}$).

(3) $$\varepsilon_{0}\left(\lambda \right)=1-{\;R}_{e}\left(\lambda \right)$$

#### 2.1.3. Reflectance (ρ)

Reflectance is the relation of radiant lux in watts between the reflected signal (${Փ}_{r}$) and the incident signal (${Փ}_{i}$) [31]. The reflectance can directly affect the interpretation of thermal values and is directly related to the angle of view of the target from the camera. Short distances from the target increase the reflectance captured by the camera; on the other hand, long distances may not be enough to obtain information from small objects.

(4) $$\rho \left(\lambda \right)=\frac{{Փ}_{r}\left(\lambda \right)}{{Փ}_{i}\left(\lambda \right)}$$

### 2.2. Experimental Environment

An experiment was conducted in the Department of Life and Environmental Sciences of the University of Tsukuba, Tsukuba, Ibaraki (36°11′19.8″ N, 140°10′20.4″ E) in the laboratory of Bioproduction and Machinery during the middle of the spring season, in which the average daily temperature range was 11–18 °C.

Japanese quail (*Coturnix japonica*) eggs were collected from a quail farm located in the city of Toyohashi, province of Aichi in Japan. The eggs were aleatory collected and shipped by mail on the same day and transported at room temperature in an appropriate package to avoid dehydration and impacts. After they arrived, the eggs were put in an airy place for approximately 5 h, and no kind of treatment, either washing or wiping, was applied. By performing the procedure, we repeated the same process performed in breeding farms. Next, we put the eggs in numerical order, marking them from 1 to 30, and the opposite face was marked from 1′ to 30′ (for each treatment of 30 eggs). The eggs were then placed directly inside the automatic incubator machine.

The equipment chosen to incubate the eggs was a fully automatic incubator machine (no brand) with 110v and automatic control of temperature, humidity, and rotation. The temperature was set to 37.8 °C with a low variation of ±0.3 °C, and the humidity was maintained at 60%, varying by ±10%.

We divided our experiment into two phases. In the first phase, a total of 60 quail eggs were incubated twice, and 30 eggs were incubated at a time. In this step, the objective was to define the methodology to collect data using a thermal micro camera, such as the position of eggs and camera, including the best interval time to collect data. Phase two consisted of the experiment. Here, we incubated 120 eggs in total, divided into four groups of 30 eggs each. For three groups, the eggs were rotated in different periods: every 90 min, 6 h, and 12 h. The fourth group were composed only of unfertilized eggs bought from grocery stores. We incubated 30 of these eggs to perform an accuracy assessment of infertile eggs and applied these data to train the deep learning models.

### 2.3. Thermal Image Acquisition and Radiometric Corrections

The thermal images were collected with a thermal micro camera FLIR^®^ (Teledyne FLIR LLC, Wilsonville, Oregon, U.S.) Model VUE™ 336, 6.8 mm, with a sensor resolution of 336 × 256 pixels and a spectral band range 7.5–13.5 μm, size 2.26” (5.74 cm) × 1.75” (4.44 cm). This is a powerful camera especially designed to board unmanned aerial vehicles (UAV) and can be controlled by a smartphone app named FLIR^®^ UAS™ 2, which is provided by the same manufacturer.

**Figure 2:**
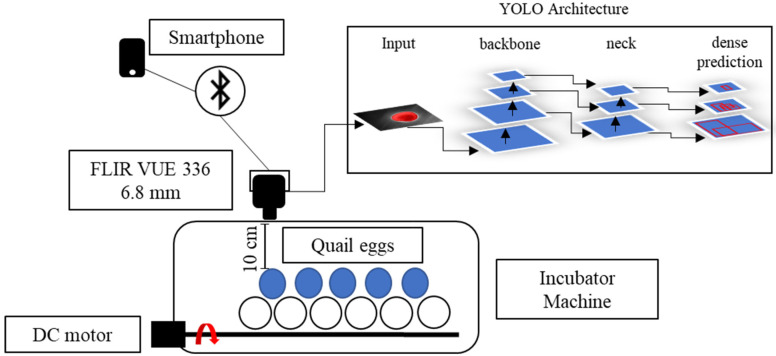
Quail egg incubation process using a thermal camera and a deep learning structure.

The camera was placed in a top-view position, with a distance of 10 cm from the targets (eggs), and the egg-by-egg images were collected inside the incubator, avoiding exposure of eggs to ambient room temperature for a long period (Figure 2). The image resolution provided by the camera was 640 pixels in width by 487 in height. Data were manually collected every 12 h (at 9 AM/9 PM), and the thermal camera was controlled by a SHARP^®^ smartphone (Sharp Corporation, Sakai, Osaka, Japan), AQUOS™ sense4 basic Model A003SH and Operational System ANDROID™ version 11 app connected by Bluetooth. This procedure was performed for 7 days for each cluster (treatment) of eggs. To collect the data, we separately moved the eggs to the left corner of the incubator, where only one egg at a time could fit in the frame image and moved the eggs by picking them up from the equator borders. Radiometric images were saved in a micro-Secure Digital (SD) Card on the camera in Joint Photographic Experts Group (JPG) format and transferred to a personal computer (PC) for data analysis. On the 8th day, the eggs were broken to assess the embryos inside the eggs.

For rotation of eggs, we employed a DC 12v motor embedded in the incubator. The eggs were turned 180° every period (90 min, 6 h and 12 h) for each cluster, and the unfertile eggs were turned every 90 min. The radiometric images were corrected with the software FLIR^®^ Thermal Studio™; the images were filtered with Isotherms and classified for the above temperatures; and manual adjustments of contrast and red saturation were performed to highlight the visible features on the eggs. Isometric transformation is a radiometric pixel classification that highlights temperatures above, in the middle or below a threshold. For egg incubation analysis, we chose the isotherms above the threshold, making it possible to capture features from fertilized and unfertilized eggs.

### 2.4. Deep Learning Algorithms and Analysis Environment

YOLOv4 is embedded in the framework Darknet (neural network framework, open source written in C programming language and CUDA). This supervised learning-based algorithm uses a single CNN to extract features of images and to create a model based on a training dataset to predict objects with a certain level of accuracy and their positions on frames or pictures.

Since its release in 2016, the YOLO object detection family has gradually expanded. The fourth generation of YOLO, also referred to as the 4th version or YOLOv4, released by Bochkovskiy et al., 2020 [33], has been one of the fastest and most accurate object detection models. YOLOv4 uses the Cross Stage Partial Darknet-53 (CSPDarknet-53), which is a new backbone that is capable of enhancing CNN learning [34], the path aggregation network (PANet) and spatial pyramid pooling (SPP). These new additions were responsible for enhancing speed by 12% and accuracy by 10% compared with YOLOv3 (previous version of YOLO).

**Figure 3:**
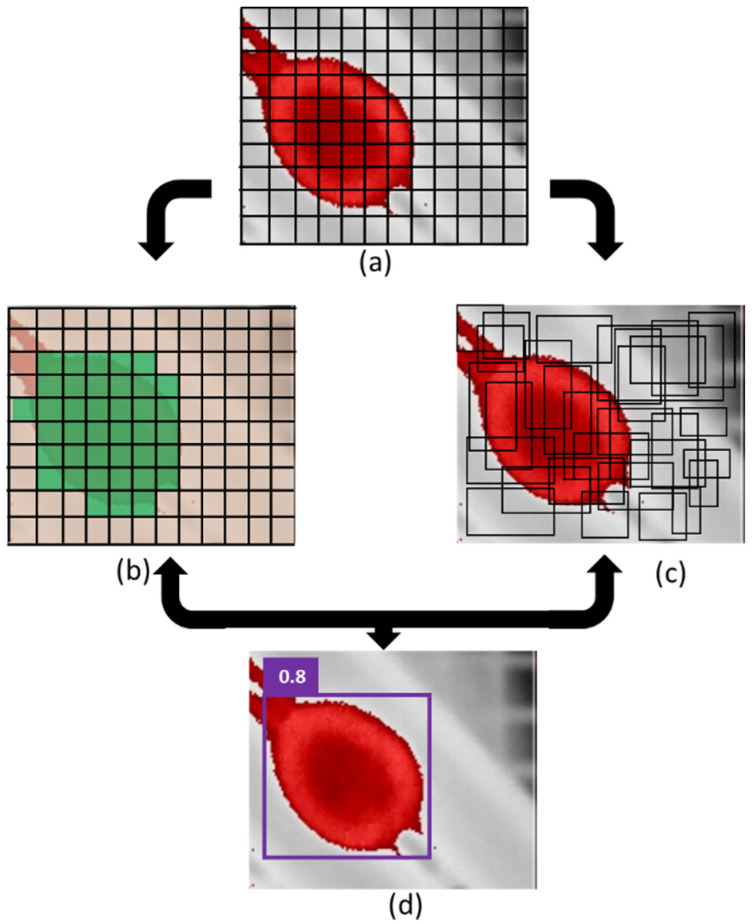
(**a**) Input frame in the deep learning process S × S grid. (**b**) Class probability map from each grid cell. (**c**) Predicted bounding box and confidence. (**d**) Final object detection.

The breakthrough of YOLO was its ability to visualize an entire image at once, dividing it into a grid of S x S and then to create a map of probabilities for Region of Interest (ROI) by regression (Figure 3). The ROI tells CNN which region has a high chance of finding the object in each frame. These characteristics were improved in YOLOv4, thus, enabling real-time object detection implementation with more accuracy. In addition, YOLOv4 was designed to be efficiently trained using only one graphics processing unit (GPU).

Recently, the YOLO series have been evolving and several versions are currently available. Improvements are being performed by companies, including YOLOv5 series [35], developed by Ultralytics^®^. However, no peer reviewed article paper has being released along with the improvements. Besides that, the community of developers engaged in the YOLO family have started to complain about the usage of YOLO‘s name by companies when launching new improved versions. YOLOv5 uses the same backbone as YOLOv4 (CSPDarknet-53), the difference is in the neck part, which is composed of a feature pyramid network (FPN) [36] and pixel aggregation network (PAN) [37], these additions are responsible for improvements in accuracy and faster training process. Another modification is that YOLOv5 is embedded in the PyTorch framework. YOLOv5 is qualified as faster and accurate, and accomplishes light files which make it suitable for low-end deployment devices.

In this work, we trained an object detection model using YOLOv4 to validate our results for embryo detection as a supportive method to assess the visual observation of thermal imaging features and to contribute to the future development of automatic classification equipment. In addition, we trained a YOLOv5-L6 and an SSD-MobileNet V2 to compare the performance of improvements on the algorithm.

YOLOv4 and YOLOv5 share the same base architecture. We also trained an SSD-MobileNet V2 model to compare the performance of different architectures when detecting unfertilized eggs using the methodology proposed in this work to collect thermal images.

SSD-MobileNet V2 [38] is another representative of one stage detector architecture that adopts the same Single Shot Detection (SSD) mechanism, similar to YOLO. However, it gained popularity due to its faster performance on low compute devices, such as mobile phones (therefore, MobileNet), and low-end computers, such as development boards NVIDIA^®^ Jetson series, Raspberry Pi and Google Coral. The V2 version of this deep learning algorithm introduced a depth-wise convolution layer, which reduces the number of parameters and contributes to improvement in the performance. The V2 added the expansion-filtering-compression, known as inverted residual structure, which contributes to improvement in its performance. In this work, we trained a model SSD-MobileNet V2 to compare the performance of the two SSD models, keeping in mind the potential application of our methodology on a high throughput system.

#### 2.4.1. Models Training

We trained all the models using only images of unfertilized eggs and collected a total of 420 images (30 images each period of 12 h). From these images, the same procedure as previously described (FLIR^®^ Thermal Studio) was performed, and then image augmentation was performed to increase the dataset to make the model more predictive.

#### 2.4.2. Data Labeling

**Figure 4:**
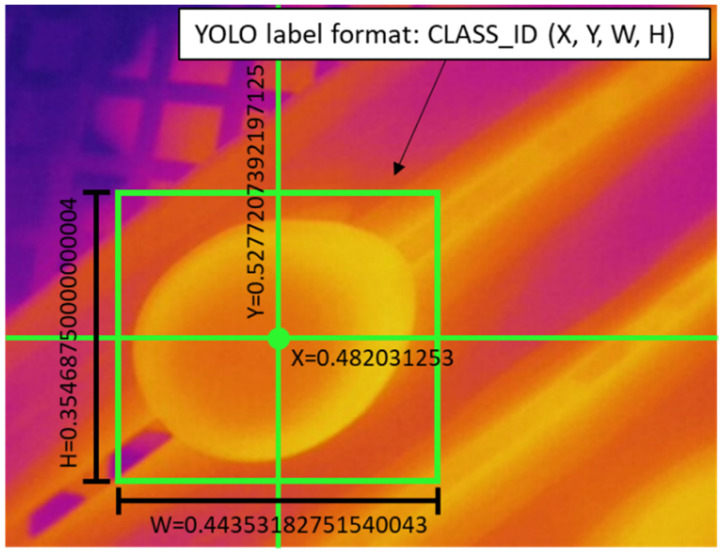
YOLO labeling format coordinates and sizes in pixels.

A short program written in Python programming language was utilized to label the images by using the OpenCV library. We drew the bounding boxes (bbox) and saved them into the YOLO format coordinates (Figure 4). As for SSD-MobileNet V2 we labeled the data using the LabelImg software in format PASCAL VOC XML.

#### 2.4.3. Data Augmentation

**Figure 5:**
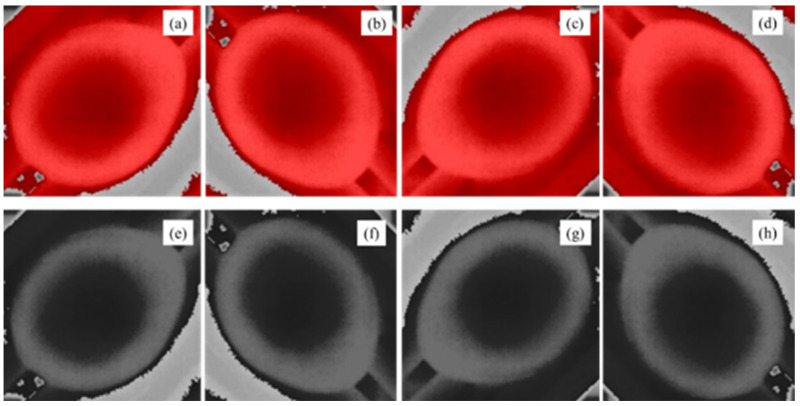
Quail egg image enhancement process and augmentation for dataset preparation during incubation in isotherm processing of original images and monochrome transformation. (**a**) Original images. (**b**–**d**). Augmentation of original images using 90°, 180° and 270° rotations. (**e**) Original position with pixel augmentation using monochrome transformation. (**f**–**h**) Augmentation of monochrome transformed images for 90°, 180° and 270° rotations.

The images from unfertilized eggs were augmented to enlarge the dataset and to make the models more effective in the detection of unseen eggs. We applied random spatial/pixel level transformations by rotating and changing the thermal effects of visualization. Thus, datasets were enhanced to 1892 images in total. Image augmentation for spatial transformation was performed in several ways: clockwise rotation 90°, clockwise rotation 180° and clockwise rotation 270° (Figure 5). In addition, pixel transformation was performed using grayscale conversion.

The images were divided into two datasets: training dataset and validation dataset. We adopted the proportion of 70:30. In total, 1325 images were selected for training and 567 images were selected for validation. To test the model, we used the data from the incubation treatment (420 images for each cluster separately tested) and then compared the precision of detection.

The network size for training the YOLOv4 model was set to 416 × 416, and the number of iterations was set to 4000 steps (Table 1). However, we stopped training when the average loss no longer decreased. The training was performed on a PC with 32 GB of RAM memory, an NVIDIA^®^ GTX 1650™ 4 GB GPU and a central processing unit (CPU) Intel^®^ Xeon™ E5-1607, Python version 3.8.5, CUDA 10.1, cuDNN 7.6.5 and OpenCV 4.4.0.

YOLOv5 model was trained in the Google Collab cloud platform. The framework version used was PyTorch 1.11.0+cu102 and 16 GB GPU Tesla T4. The training parameters are shown in Table 1. The epochs of training (number of iterations) were set to 60, the source code was cloned from official Ultralytics^®^ GitHub [35].

SSD-MobileNet V2 was trained using the same computational resources used for training YOLOv4. However, we trained the model using the TensorFlow API object detection framework, in an environment built with Tensorflow 2.3.1 and Tensorflow-gpu 2.3.1. The input size of this model was 320 × 320 and 40,000 steps for training.

#### 2.4.4. Model Evaluation

**Figure 6:**
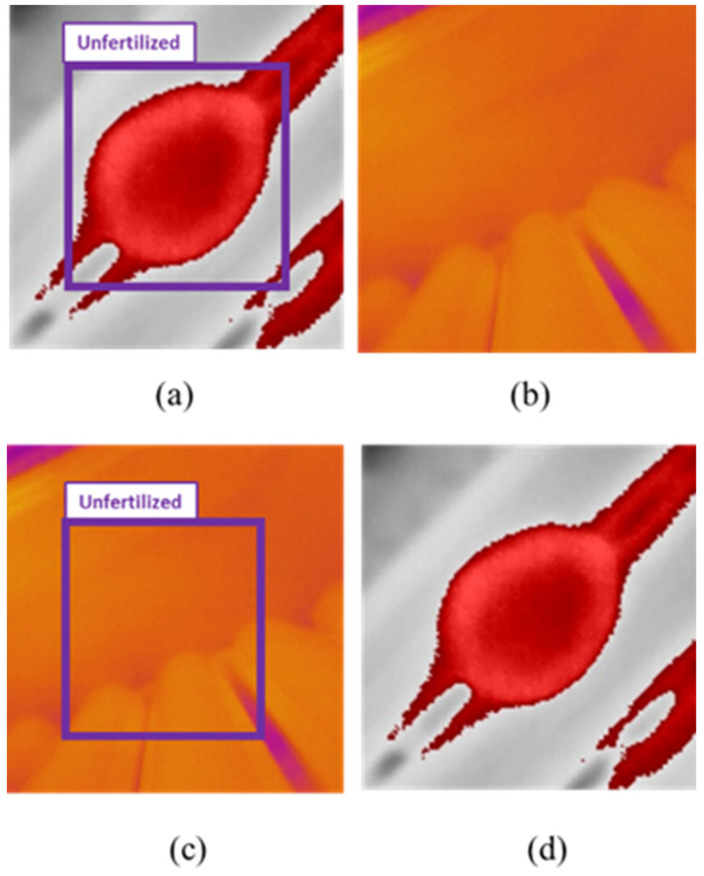
(**a**) True positive (TP); (**b**) true negative (TN); (**c**) false-positive (FP); (**d**) false negative (FN).

Several metrics were applied to evaluate the deep learning models, including precision (P), recall (R), F1 score and mean average precision (mAP). Object detection evaluations are based on 4 factors: true positive detections (TP); unfertilized eggs are correctly detected; true negatives (TN); unfertilized eggs are not shown and are not detected; false-positive (FP); object is detected, but it does not correspond to any class, and false negative (FN): model does not label an object but was supposed to perform this task (Figure 6).

P (Equation (5)) and R (Equation (6)) are measurements that evaluate the relevance of detection. R returns the real relevance of the results, considering false negative detections, while P considers false-positive detections. The F1 score (Equation (7)) is a measurement that indicates the relation degree of P and R, as the higher the F1 score is, the higher the values of P and R, the more accurate the detections may be.

**Figure 7:**
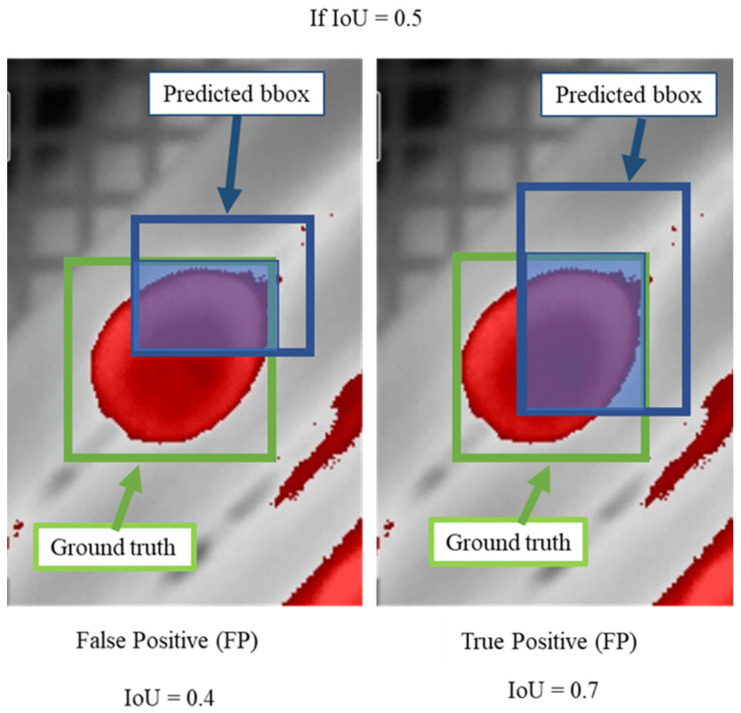
IoU details of false-positive and true positive detection for unfertilized eggs.

To categorize truthfulness of a class, the concept of intersection over union (IoU) was applied (Equation (8)). This metric regards ground truth and detection (Figure 7).

(5) $$P=\frac{\mathrm{TP}}{\mathrm{TP}+\mathrm{FP}}$$

(6) $$R=\frac{\mathrm{TP}}{\mathrm{TP}+\mathrm{FN}}$$

(7) $$F1\;\mathrm{score}=\frac{2\mathrm{PR}}{P+R}$$

(8) $$\mathrm{IoU}=\frac{\mathrm{area}\;\mathrm{of}\;\mathrm{overlap}}{\mathrm{area}\;\mathrm{of}\;\mathrm{union}}$$

The mean average precision (mAP) is the average precision (AP) over the number of classes (Equation (10)). The AP is a metric used in the PASCAL VOC challenge [39]; it was obtained by calculating the area under the P-R curves interpolated at 11 points (Equation (9)). However, the interpretation may vary depending on the problem of classification. For instance, in COCO dataset evaluation, the metric AP was considered equivalent to mAP. This metric is important for object detection model evaluation because it considers the arrangement between P and R and the relation of FP and FN.

(9) $$\mathrm{AP}=\frac{1}{11}\sum_{R_{i}}{\mathrm{PR}}_{i}$$

(10) $$\mathrm{mAP}=\frac{1}{N}\sum_{i=1}^{N}{\mathrm{AP}}_{i}$$

## 3. Results

The first two trials served the purpose of establishing the best procedure to collect data for the experiment. The results from this trial showed that collecting data from the upside face of eggs was more effective for the instant identification of features than collecting data from the downside face at either the short end or the large end of eggs. From the first trial, we collected images by removing the eggs from the incubator machine. In the second trial, we collected images from the inside of the incubator machine, being best for preventing instantaneous exchange of temperature within the environment and for improving thermal transmission. On the 8th day, the eggs were artificially hatched to assess the presence of embryos (Table 2). As expected, the unfertilized eggs presented no embryos.

### 3.1. Thermal Features of Incubating Eggs

**Figure 8:**
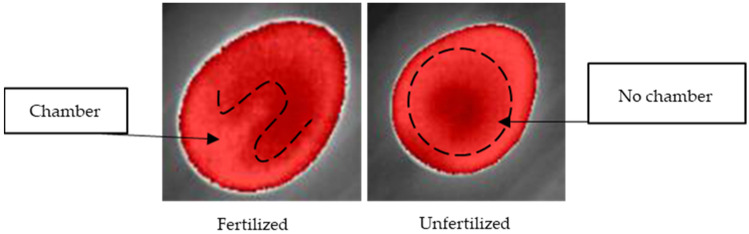
Isotherm filtering for observing different feature patterns of fertilized (No. 10) and unfertilized eggs (No. 11) during the first days of incubation.

Using isotherm filtering, we observed different feature patterns of fertilized eggs to unfertilized eggs (Figure 8).

**Figure 9:**
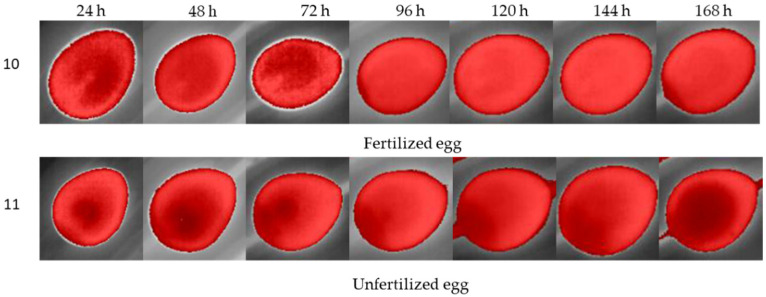
Thermal analysis under isotherm classification for fertilized and unfertilized eggs after turning for a 12 h period over a 7-day incubation period.

Different periods of egg turning showed different patterns of thermal images, although similarities were also observed. For example, the common characteristic of most fertilized eggs was the presence of a “chamber” structure in the middle of the dark red spot (which is related to the composition of embryo and egg structures). As the days of incubation passed, the chamber structure became less apparent (Figure 9). For example, through thermal analysis under isotherm classification, the evolution of two eggs over 7 days is shown. The eggs were incubated for a turning period of 12 h. It is possible to observe that after 72 h, the structures became less apparent compared with unfertilized eggs. For unfertilized eggs, the dark spots became larger due to the accumulation of gases from the decomposition of yolk and dehydration.

**Figure 10:**
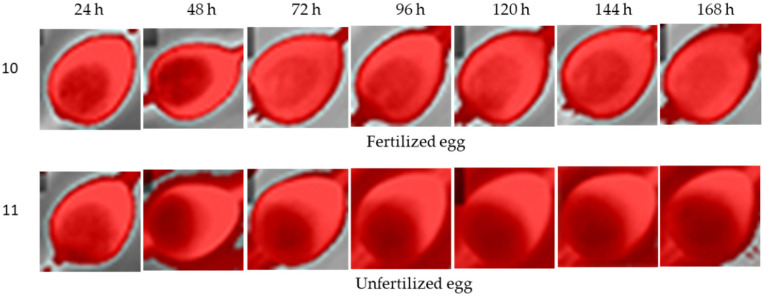
Thermal images or eggs incubated at turning periods of 6 h in a total 7-day incubation period for fertilized and unfertilized eggs.

Eggs turned every 6 h showed constant features during the data collection period (Figure 10). Fertilized eggs had chamber features, while the binary pair did not show the same characteristics as eggs incubated for the 12 h rotation period.

**Figure 11:**
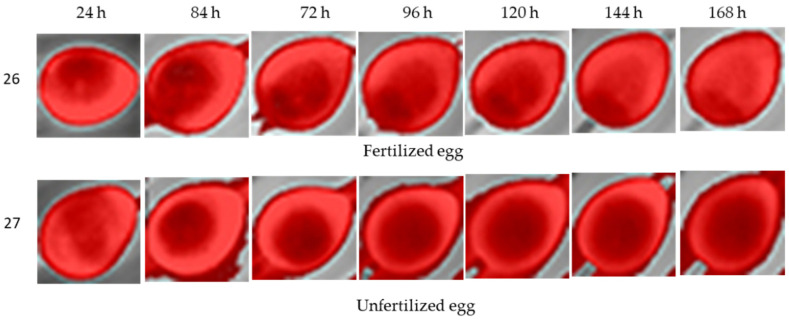
Thermal images from eggs incubated at turning periods of 1.5 h in a total 7-day incubation period for fertilized and unfertilized eggs.

For the eggs incubated during the 1.5 h period of turning, we observed that similar structures were also identified; however, from visual analysis by human eyes, such features were less clear than those of the previous 2 images shown above (Figure 11).

### 3.2. Training Results

**Figure 12:**
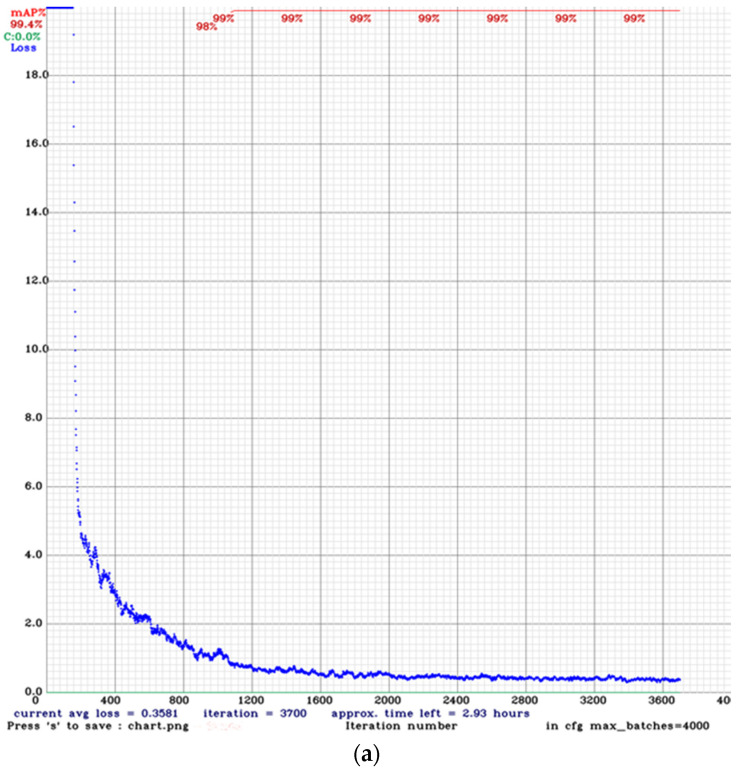

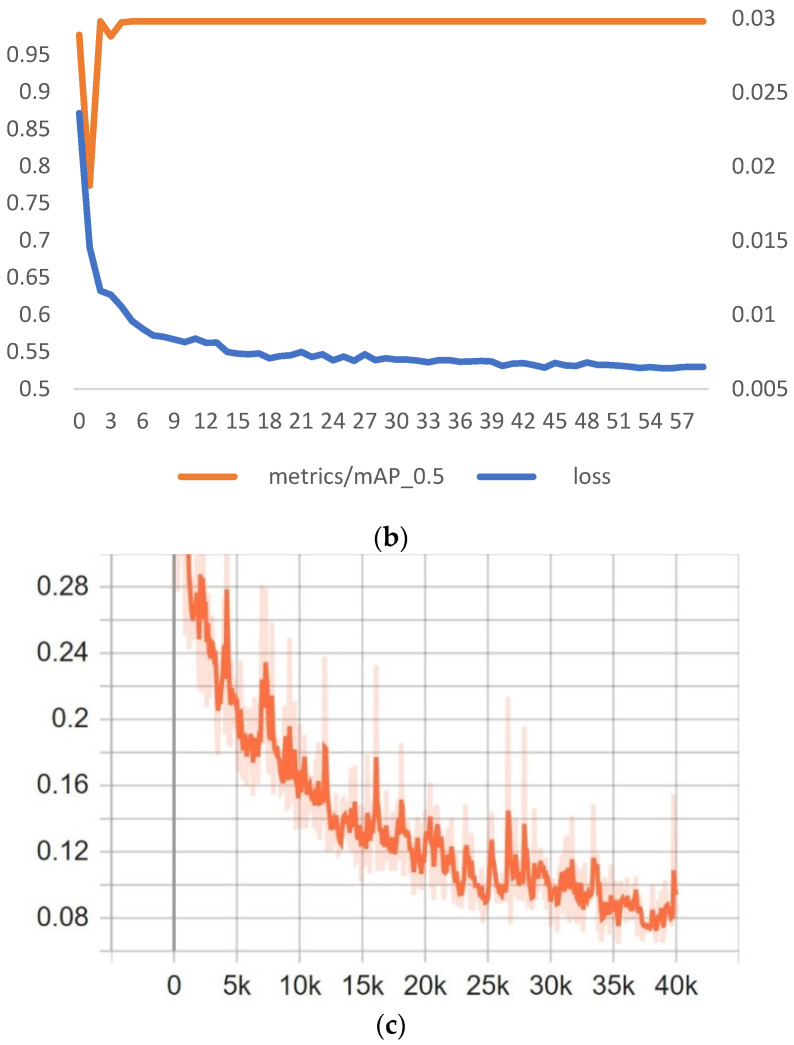
(**a**) Average loss during training of datasets using Darknet framework for YOLOv4. (**b**) YOLOv5 Training in Google Collab using PyTorch framework. (**c**) SSD-MobileNet V2 training loss in the TensorFlow framework.

The YOLOv4 model was trained for approximately 20 h, and we stopped the training when the average loss reached less than 0.5 and the mAP was higher than 90% for the training dataset (Figure 12a). YOLOv5 trained in Google Collab took 1 h to be completed (Figure 12b). The SSD-MobilenetV2 model was obtained after 4 h of training in the PC environment (Figure 12c).

**Figure 13:**
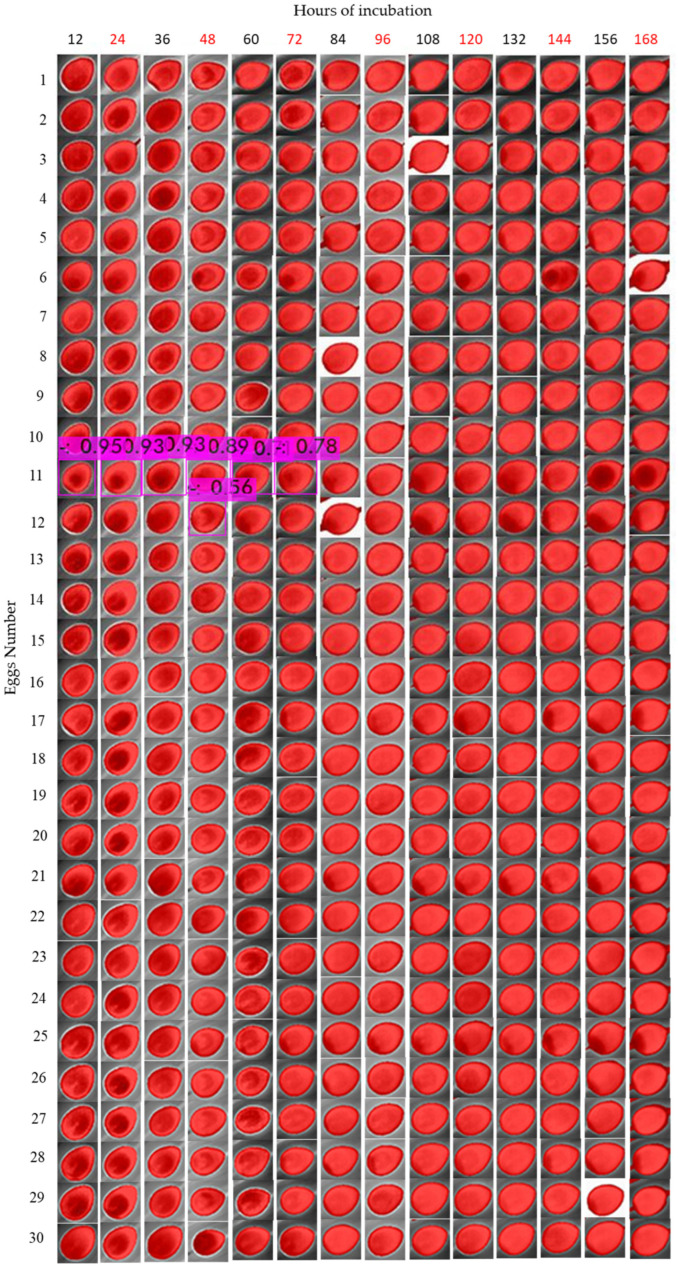
Object detection of eggs turned every 12 h under no threshold.

**Figure 14:**
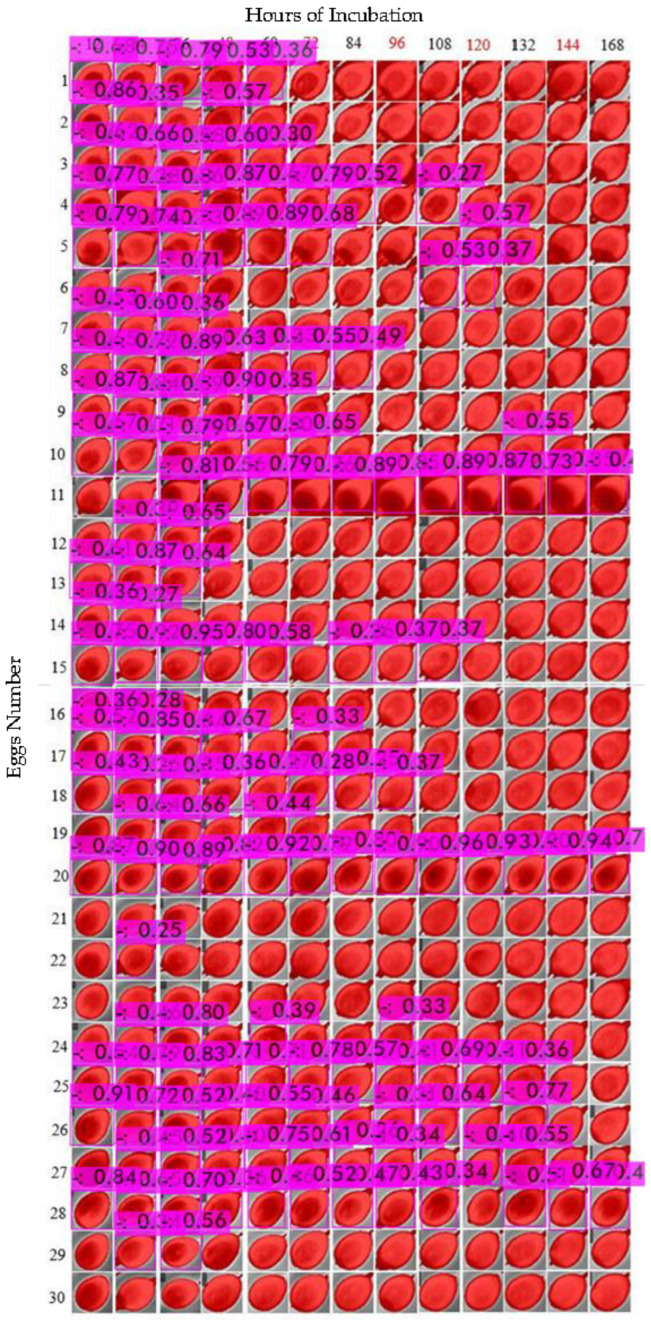
Object detection of eggs turned every 6 h under no threshold.

**Figure 15:**
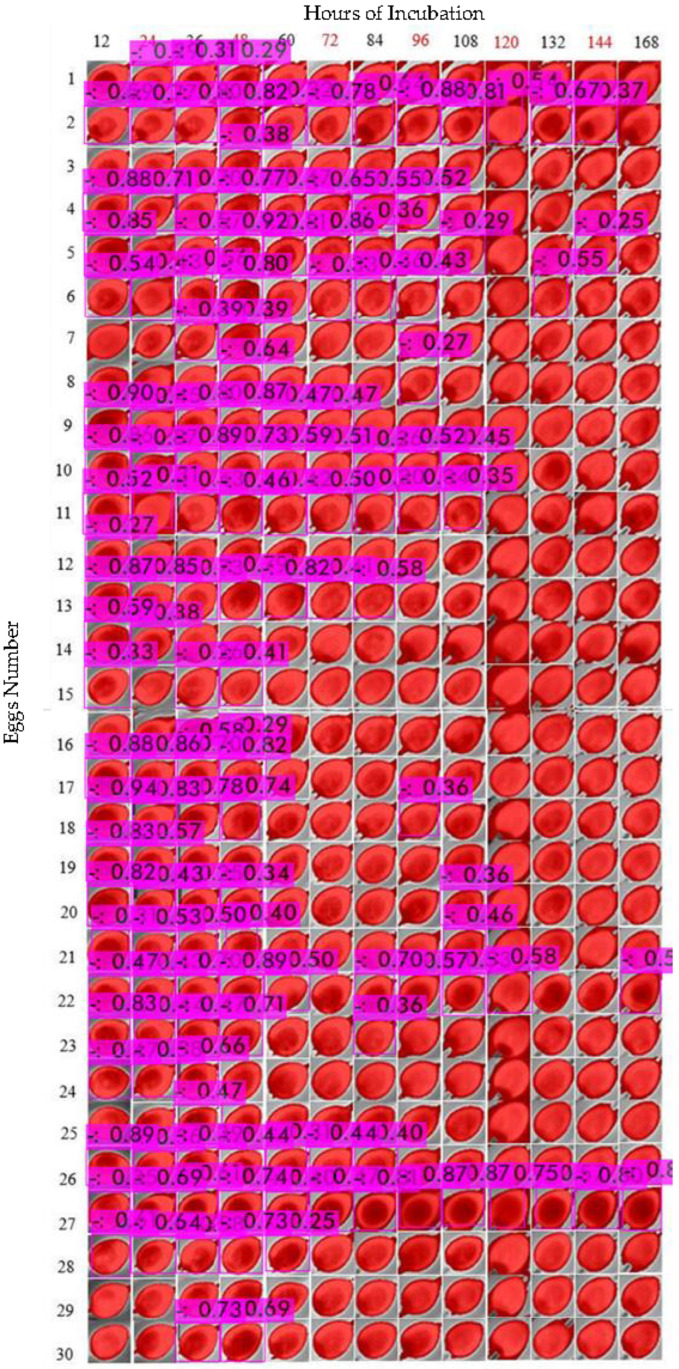
Object Detection of eggs turned every 1.5 h (90 min) under no threshold.

The evaluation of training was performed by calculating the measurements as described in the results (Table 3). To test the model on 3 experimental treatments, we composed all the data of each cluster in only one image. As shown in the table, each egg picture was cropped to 30 × 30 pixels to reduce the size for comparing images from 12 h, 6 h and 1.5 h (Figure 13, Figure 14 and Figure 15 show an example of testing YOLOv4 model).

**Figure 16:**
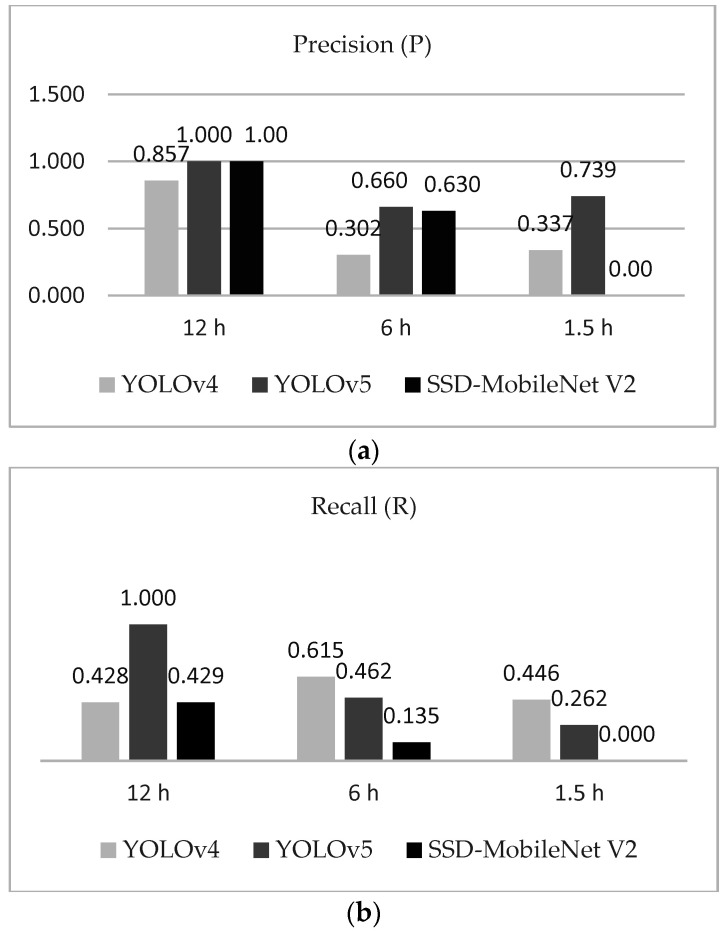

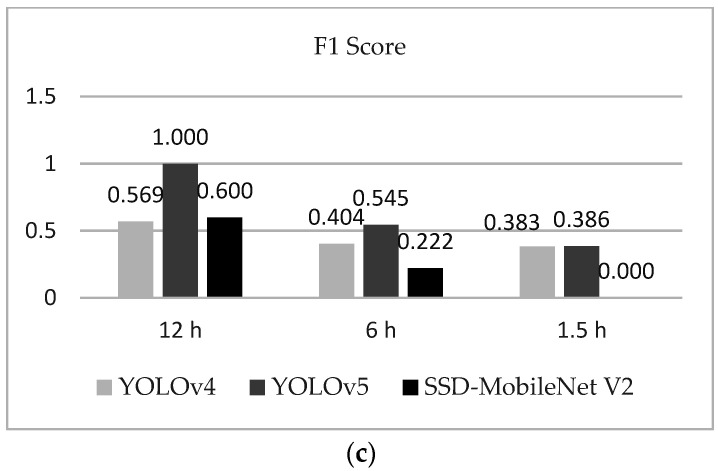
(**a**) Testing data set Precision comparison between 3 models. (**b**) Recall comparison. (**c**) F1 Score comparison.

To compare the images, we numbered the eggs from 1 to 30 in the rows. For the columns, we indicated the periods of data collection, from 12 h of incubation (first day) to 168 h (seventh day). Next, we calculated the precision, recall and F1-score according to the detection assessment (Table 4 and Figure 16). Note that we discarded the “156 h” data from the 6 h and 1.5 h datasets, due to errors generated while collecting images. However, the analysis was conducted based only on the presented data as follows.

Table 3 shows the deep learning algorithm’s training performance, according to the metrics explained in the Section 2.4.4. The mAP@0.50 was the main evaluation method for training models, and can be interpreted as the area under the interpolation of the P and R curves. The value 0.50 concerns the IoU, that considers an object detection if the overlap section overcame 50% of the ground truth (labeled object). YOLOv5 showed better mAP@0.50, followed by YOLOv4 and SSD-MobileNet V2.

The unfertilized eggs were assessed on the 8th day of incubation, as indicated in Table 2. The calculation presented in Table 4 and Table 5 are based on that.

In Table 4 the object detections resulting from our test dataset were tabulated. The total detections show the number of bounding boxes that were displayed for each model and for each treatment for detection of unfertilized eggs. The 6 h dataset displayed more bounding boxes over the 3 datasets. The total detection could also be interpreted as the sum of TP and FP detections, according to the example given in Figure 6, Section 2.4.4. The TP column stands for the bounding boxes displayed around correct unfertilized eggs. TN refers to the total of eggs that were neither classified as unfertilized eggs nor having the probability of being an unfertilized egg falling below the threshold (50% for YOLOv4 and YOLOv5 and 18% for SSD-Mobilenet V2), and, therefore, not detected as unfertilized eggs. FN is the quantity of unfertilized eggs that were not detected by the object detection algorithms. FP represents the number of bounding boxes displayed around eggs but not corresponding to unfertilized eggs.

Table 5 results from the calculation of metrics by using the data on Table 4. The P value was obtained using Equation (5), and can be understood as the relation of unfertilized eggs that were correctly marked with a bounding box over the total bounding boxes. The R is a metric that indicates the proportion of unfertilized eggs that were not detected by the object detector but should have been detected. The F1 score stands for the balance between P and R, and from this metric we could evaluate the proportion of assertiveness of each deep learning model on the unseen dataset. In our case, the F1 score could tell us the proportion of unfertilized eggs detected by the YOLOv4, YOLOv5 and SSD-MobileNet V2 algorithms.

## 4. Discussion

The embryo development of quail eggs takes 16.5 days to complete according to Ainsworth et al., 2010 [40]. The same author also affirms that although the incubation period of quail eggs and hen eggs differ (21.5 days for hen’s eggs), the embryo development stages are quite similar, making it possible to compare the development process between them. Several studies on the characterization of avian embryo development have been reported [41,42,43,44]. Most of the studies were focused on understanding the biological functioning of structures and genetics. However, due to the recent availability of sophisticated sensors and computational methods, new approaches for poultry yield improvements have been reported. In this study, we aimed to unleash the potential of thermal cameras to detect features that could differentiate fertilized eggs from unfertilized eggs, as a nondestructive and noncontact technique. The overall goal was to contribute to monitoring the hatching process for more efficient quail hatching management.

The method of incubation utilized in this study differed from most common automatic incubator machines in industrial quail farms. For industrial incubators, the eggs are usually allocated in the vertical position and are periodically turned 45°, which is specifically related to facilitating labor operations on a large scale. Van de Ven et al., 2011 [45] compared the position of hen eggs during incubation and concluded that position did not significantly interfere with the hatching rate. Oliveira et al., 2020 [28] compared different turning periods of hen eggs and concluded that reducing the frequency of turning eggs affects the hatchability of chicks. In this study, we did not evaluate the hatchability of eggs or mortality.

Thermal cameras produce images by solving the intensity of infrared wavelengths transmitted to the thermal sensor; the major limitations of this kind of camera are low resolution and high price [19]. Reflectance, transmittance, and emissivity are the main factors of the application of thermal cameras, and the main use of this kind of sensor is specifically related to nocturnal vision and body temperature monitoring. In this study, we proposed the use of thermal imaging to observe the thermal behavior of quail eggs during incubation stages. The use of isotherm filtering was the main point of this proposed methodology to identify unfertilized eggs, as isotherms can cluster radiometric information under a threshold. The variations in the eggshell, due to the development of the embryo and the transformation of the yolk sac content, allantois and air chamber, were expected to interfere with the dynamics of gases through the micropores of the shell. Therefore, thermal imaging could capture these changes. However, the lack of a standard configuration of isotherm controls might be the main factor responsible for the poor clarity of features that could facilitate the classification of embryo absence.

The deep learning-based algorithms have proved to be a powerful tool for vision systems operation in real time, and the high precision of the model showed that it is possible to extract features of thermal images from unfertilized eggs. However, we observed that more data were necessary to improve the robustness of the model. We realized that classifying images using only one class might not be enough to push the bias of the model for more precise detailed feature extractions inside the eggs. In further research, we will address overcoming such limitations by increasing the classes.

From the results, we observed that YOLOv5 outperformed all the other models tested. For training performance, mAP@0.50 was smoothly of a higher standard than the other two models, and the F1 score in the testing dataset was better, which was because the model presented less FP over all detections. YOLOv4 followed the YOLOv5 and the worst results were obtained from SSD-MobileNet V2. The result of P showed that YOLOv5 was more precise for detecting unfertilized eggs under 50% of confidence.

We also noticed that the fertilized egg thermal profile showed similar structures that were not dependent on the period of egg turning. However, the low resolution of thermal cameras can compromise the recognition of features; that is, the distance and transmittance effects can compromise the detection of features. We observed that errors occurring during the collection of data may be responsible for poor radiometric data, and consequently, the misclassification of eggs. For the testing results, we observed that a few unfertilized eggs in each treatment were not sufficient to infer that different turning periods could improve the detection of a class. However, using these datasets, we could test the deep learning model and ensure the potential for detecting embryos (by exclusion of unfertilized eggs) in the early stages of incubation. The majority of unfertilized eggs were correctly recognized, although the error caused by false-positive detections led the precision of the model to low values (with the exception of the 12 h intervals, which presented a low error, but only one fertilized egg).

This work proved that it is possible to use a nondestructive and noninvasive method to identify embryos in the early stages of development. In addition, thermal cameras can contribute to an enhanced hatching rate because unfertilized eggs can be removed in early incubation, which represents a gain in overcoming waste of resources, such as space and energy in quail breeding farms. High throughput applications of the methodology proposed in this work are possible. However, fine studies will be addressed for standardization of isotherms filtering, as the lack of filtering pattern represents the main bottleneck for the methodology presented herewith. The breakthrough methodology proposed to identify embryos in early stages during incubation period can be extended to other avian eggs. Furthermore, low-cost devices and online inference are possible as well. Further studies will be conducted in this regard by expanding data sets and developing fine-tuning kernels for fertilized eggs thermal imaging.

## 5. Conclusions

The methodology developed in this study was able to identify different features of fertilized quail eggs from unfertilized eggs by using a thermal camera and deep learning algorithms. The use of isotherm analysis during the incubation period, associated with the deep learning object detection algorithms, showed immense potential to compose automated systems based on CV for the classification of unfertilized eggs in early stages. Different characteristics for fertilized eggs and unfertilized eggs were identified for all the evaluated treatments, and it was concluded that different periods of egg rotation do not interfere in the identification of embryos in early incubation. We noticed that unfertilized eggs could be identified after 12 h of incubation. To test the model, we reduced the original size and clustered all images into only one image dataset to test the robustness of the model when fitting the model all at once. The results showed that the model did not overfit the training dataset, as we could observe that most of our targets were correctly classified. The results from the evaluation of the models showed high mAP for the validation dataset. However, when testing the images under each treatment, the precision was reduced. Several reasons may be responsible for the reduced precision when testing the models, such as the short resolution of testing images and issues associated with errors when collecting data using a thermal camera. A further study will be conducted to enhance the precision of detection, including enlargement of the dataset and classification of fertilized eggs.

## Figures and Tables

**Table 1:** YOLOv4 and YOLOv5 training parameters.

	Batch	Input Size	Momentum	Decay	Learning Rate
YOLOv4	64	416 × 416	0.949	0.0005	0.0001
YOLOv5	16	416 × 416	0.937	0.0005	0.0001

**Table 2:** Assessment of embryos on the 8th day of incubation.

Period of Turning Eggs	With Embryo	No Embryo	N° of Egg w/No Embryo
12 h	29	1	11
6 h	26	4	1, 11, 20, 28
1.5 h	25	5	2, 8, 14, 22, 27
Unfertilized 1.5 h	0	30	1–30

**Table 3:** YOLOv4, YOLOv5 and SSD-MobileNet V2 training model evaluation using the IoU threshold of 0.5 or 50% for the detection of unfertilized eggs.

Model	Dataset	Precision (P)	RecaIl (R)	F1 Score	Average IoU (%)	mAP@0.50 (%)
YOLOv4	Validation	0.97	0.99	0.98	77.39	98.62
YOLOv5	Validation	1	0.99	0.99	- *	99.5
SSD-MobileNet V2	Validation	1	0.94	0.96	- *	91.8

* Information not available.

**Table 4:** Testing dataset detection evaluation parameters under a threshold of 0.5 in the YOLOv4 and YOLOv5, SSD-MobileNet V2 the threshold at 0.18 models for the detection of unfertilized eggs.

Model	Dataset	Total Detections	True Positive (TP)	True Negative (TN)	False Negative (FN)	False Positive (FP)
YOLOv4	12 h	7	6	413	8	1
	6 h	106	32	314	20	74
	1.5 h	86	29	334	36	57
YOLOv5	12 h	11	11	409	0	0
	6 h	36	24	384	28	12
	1.5 h	23	17	397	48	6
SSD-MobileNet V2	12 h	6	6	414	8	0
	6 h	11	7	409	45	4
	1.5 h	6	0	414	65	6

**Table 5:** Evaluation of Precision (P), Recall (R) and F1-score in the YOLOv4 and YOLOv5 models for the detection of unfertilized eggs.

Model	Dataset	Precision (P)	Recall (R)	F1-Score
YOLOv4	12 h	0.857	0.428	0.569
	6 h	0.301	0.615	0.404
	1.5 h	0.337	0.446	0.383
YOLOv5	12 h	1	1	1
	6 h	0.66	0.5	0.56
	1.5 h	0.60	0.26	0.36
SSD-MobileNet V2	12 h	1	0.42	0.59
	6 h	0.63	0.13	0.21
	1.5 h	0	0	0

## Data Availability

The dataset that was generated and analyzed during this study is available from the corresponding author upon reasonable request, but restrictions apply to the data reproducibility and commercially confident details.
